# Supplementary material for: Sulfatide Binds to Influenza B Virus and Enhances Viral Replication
Source: Viruses. 2025 Apr 5;17(4):530. doi: 10.3390/v17040530 (PMC12031359; doi:10.3390/v17040530)
Supplement: Supplementary file 1 [file viruses-17-00530-s001.zip › viruses-3539114-supplementary.pdf]

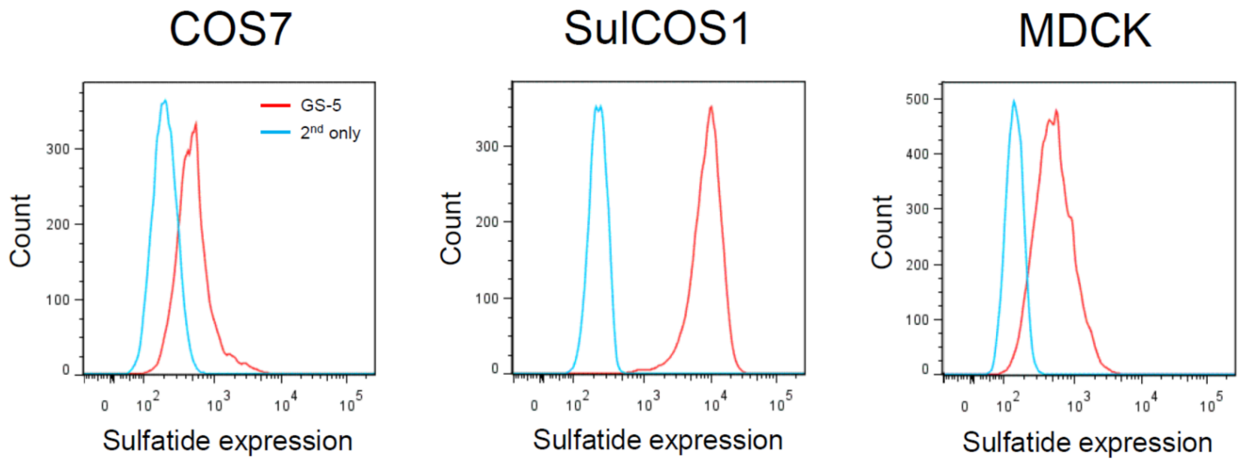

Figure S1. Flow cytometric analysis of sulfatide expression on COS7, SulCOS1, and MDCK cells. Suspension cells were incubated with anti-sulfatide mouse IgM antibody (GS-5) followed by PE-labeled anti-mouse IgM (Red). The negative control was cells incubated only with the secondary antibody (2nd only) (Blue). Cell fluorescence was analyzed using a flow cytometer FACSCelesta and FlowJo software. The X-axis represents the extent of sulfatide on the cell surface, and the Y-axis represents cell counts.

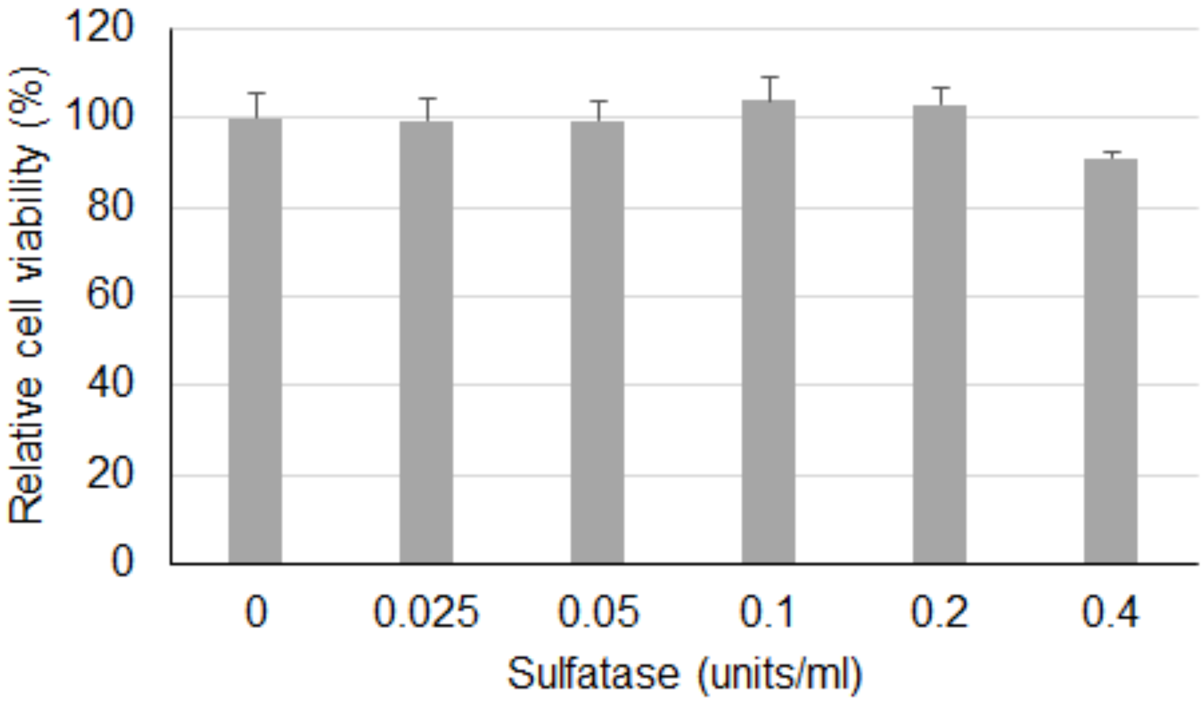

Figure S2. Cytotoxicity analysis of sulfatase treatment. MDCK cells were plated onto a 96-well plate and incubated with sulfatase from *Aerobacter aerogenes* (0.025 ~ 0.4 units/ml) at 37°C for 24 hours. Cell viability was measured using Cell Counting Kit-8 (Dojindo). No statistically significant differences were detected across treatments by Tukey's test.

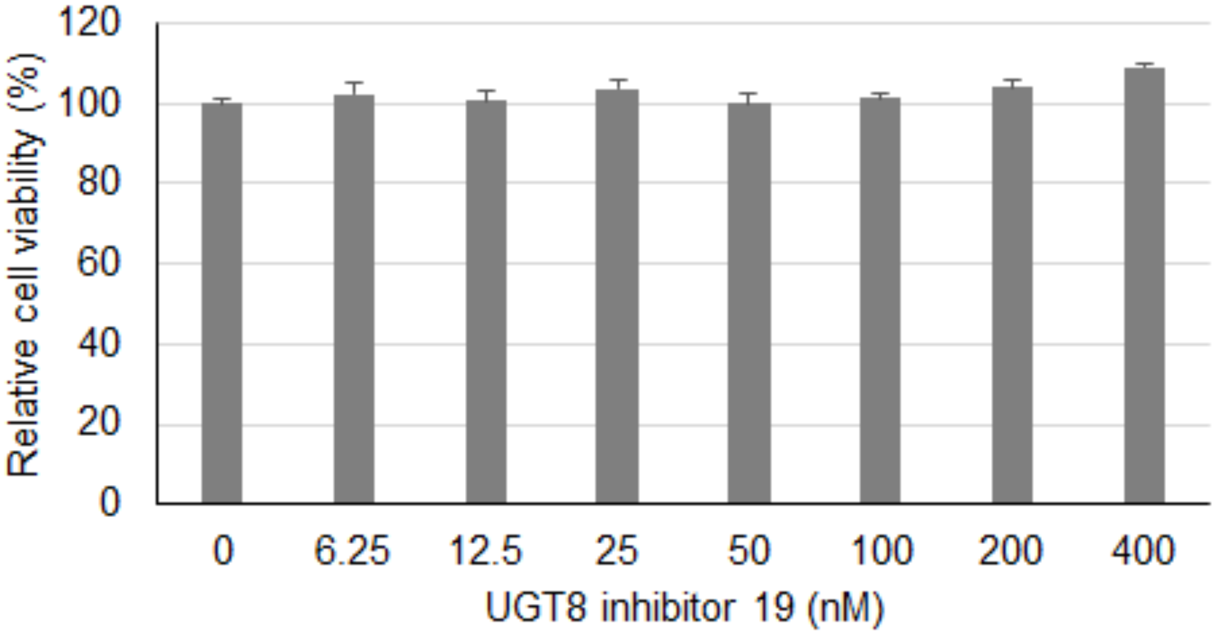

Figure S3. Cytotoxicity analysis of UGT8 inhibitor 19. A549 cells were plated onto a 96-well plate and incubated with UGT8 inhibitor 19 (6.25~400 nM) at 37°C for 48 hours. Cell viability was measured using Cell Counting Kit-8 (Dojindo). No statistically significant differences were detected across treatments by Tukey's test.
